# Seabuckthorn Seed Meal Protein-Based Inhibitory Peptides Targeting Multiple Hyperglycemic Enzymes: Optimization of Process and Probing of Mechanisms

**DOI:** 10.3390/foods14111876

**Published:** 2025-05-26

**Authors:** Qi Shan, Yeping Jia, Tonghua Wu, Jun Zhang, Liang Shan

**Affiliations:** 1 School of Food Science and Technology, Jiangnan University, Wuxi 214122, China; 6220112130@stu.jiangnan.edu.cn (Q.S.); 6220111047@stu.jiangnan.edu.cn (Y.J.); 2 Xinjiang Engineering Technology Research Center for Seabuckthorn Fine & Deep Processing, Xinjiang Zhongke Seabuckthorn Technologies Co., Ltd., 68 Jialangqi Road, Aheqi 843500, China

**Keywords:** seabuckthorn seed meal protein, hypoglycemic peptides, *α*-glucosidase, dipeptidyl peptidase-IV, molecular docking

## Abstract

This work utilized seabuckthorn seed meal protein (SSP) to develop hypoglycemic peptides via controlled protease catalyzed hydrolysis. Among the SSP hydrolysates (SSPHs) obtained by means of various proteases, the SSP hydrolyzed by dispase (SSPD) exhibited extraordinary inhibitory abilities against three key enzymes involved in glucose metabolism: α-glucosidase, α-amylase, and dipeptidyl peptidase-IV (DPP-IV). Following process optimization and purification, SSPD displayed remarkable inhibitions to *α*-glucosidase (IC_50_: 3.45 ± 0.18 mg/mL) and DPP-IV (IC_50_: 5.01 ± 0.21 mg/mL), respectively. Molecular docking analysis and in vitro verification revealed three peptides in the SSPD with *α*-glucosidase inhibition: FHF, FFI, and FGI (IC_50_: 3.98 ± 0.16 mM, 8.21 ± 0.21 mM, 11.57 ± 0.20 mM), and three peptides with DPP-IV inhibition: IYF, IGF, and LFF (IC_50_: 5.32 ± 0.15 mM, 7.17 ± 0.14 mM, 7.62 ± 0.19 mM). These findings demonstrate that SSP holds promise as a significant natural resource for the creation of multifunctional hypoglycemic peptides, which can be utilized in nutritional and functional food applications.

## 1. Introduction

Seabuckthorn (*Hippophae rhamnoides* L.), a widely grown plant across temperate regions of Eurasia, is recognized for its impressive medicinal and nutritional properties [1,2]. This plant is a significant source of numerous nutrients and bioactive compounds, which include essential vitamins, fatty acids, amino acids, flavonoids, and superoxide [3]. These bioactive constituents are linked to a wide range of health benefits, such as blood pressure regulation, anti-inflammatory and antidiabetic effects, and cardiovascular protection [4]. Seabuckthorn seed meal, a residual by-product of oil extraction, is composed of more than 35% protein and provides a complete amino acid profile [5,6], which positions it as a valuable protein source with potential for further development and utilization. However, it is currently used as livestock feed, which leads to the underutilization of its valuable protein content [7].

Diabetes is a significant health concern, reaching alarming levels in contemporary society. Over the past five decades, the prevalence of diabetes has steadily increased, with an estimated 425,000 new cases reported annually [8]. Projections indicate that the prevalence of diabetes may increase by over 50% by 2045, potentially impacting around 693 million individuals [9]. Reducing or inhibiting the key glucose metabolism enzymes, such as *α*-glucosidase, *α*-amylase, and dipeptidyl peptidase-IV (DPP-IV), is an effective diabetes treatment strategy [10]. Inhibiting *α*-glucosidase and *α*-amylase slows the conversion of complex carbohydrates into absorbable monosaccharides, thus reducing postprandial glucose levels [11]. Meanwhile, the suppression of DPP-IV activity can preserve incretin hormones such as GLP-1 and GIP, thereby enhancing insulin secretion and lowering blood glucose concentrations [12]. Peptides derived from plant proteins through enzymatic hydrolysis have attracted increasing attention as natural inhibitors of hyperglycemic enzymes. Thus far, numerous studies have reported on natural peptides, such as *α*-glucosidase or DPP-IV inhibitors derived from black beans, buckwheat, oats, and highland barley hydrolysates [13,14]. However, few attempts, whether using SSP or targeting multiple hyperglycemic enzymes, were reported on developing such inhibitory peptides.

This study aimed to systematically evaluate the hypoglycemic potential of SSP hydrolysates (SSPHs) obtained by various proteases, with a particular focus on their inhibitory ability against α-glucosidase, α-amylase, and DPP-IV. We successfully identified a hydrolysate exhibiting simultaneous inhibitory effects against *α*-glucosidase, *α*-amylase, and DPP-IV, while optimizing the hydrolysis conditions based on *α*-glucosidase and DPP-IV inhibition as indices. Through peptidomics and molecular docking analyses, we examined the mechanisms underlying the inhibition of *α*-glucosidase and DPP-IV activity, providing a foundation for the development and application of natural hypoglycemic peptides. Meanwhile, the study also highlights the potential for reducing waste of SSP and enhancing the value of derived products from SSP.

## 2. Materials and Methods

### 2.1. Materials

Seabuckthorn seed meal was sourced from Aheqi County, Kizilsu Kirgiz Autonomous Prefecture, Xinjiang Uygur Autonomous Region, China. Alpha-glucosidase (yeast), alkaline proteinase, compound proteinase, dispase, bromelain, papain, *p*-Nitrophenyl-β-D-Glucopyranoside (*p*-NPG), Gly-Pro-pNA, and 3, 5-Dinitrosalicylic acid (DNS) were procured from Shanghai Yuanye Bio-Technology Co., Ltd. (Shanghai, China). DPP-IV (human) and *α*-amylase (porcine pancreas) were sourced from Sigma-Aldrich (Shanghai, China). Soluble starch was acquired from Shanghai Titan Scientific Co., Ltd. (Shanghai, China). All other reagents used were of analytical purity.

### 2.2. Extraction of SSP

SSP was extracted following the alkali-solution and acid-isolation approach [15]. Seabuckthorn seed meal was mixed with distilled water at a ratio of 1:15 (*w*/*v*) and the pH was maintained at 10.0 by 1 M NaOH. The mixture was stirred at 40 °C for 1 h, followed by centrifugation at 4000 rpm for 20 min to collect the supernatant. The pH of the supernatant was then lowered to 4.0 with 1 M HCl to induce protein precipitation. After resting for 30 min, the solution was centrifuged again, and the precipitate was washed with distilled water to neutral. The resulting protein was re-dissolved, frozen, and subsequently freeze-dried to obtain SSP.

### 2.3. Preparation of SSPHs

Prior to hydrolysis, each SSP suspension (4% *w*/*v*) was preheated at 85 °C for 30 min to induce protein denaturation, thereby enhancing hydrolysis efficiency [16]. Subsequently, hydrolysis was conducted with an enzyme to a protein ratio of 8400 U/g using five proteases, each at its optimal temperature and pH [17], as follows: (1) Alkaline proteinase: pH 10.0, 40 °C; (2) Compound proteinase: pH 7.5, 50 °C; (3) Dispase: pH 7.5, 40 °C; (4) Bromelain: pH 7.5, 50 °C; (5) Papain: pH 7.5, 40 °C. Following hydrolysis for 3 h, the solution was heated at 85 °C for 15 min to deactivate the proteases, after which the pH was adjusted to neutral.

Finally, SSPHs were obtained from the centrifuged supernatants. SSPHs were stored at 4 °C prior to analyses and activity assessments.

### 2.4. Degree of Hydrolysis (DH)

DH was determined by the pH-stat method [18]:

DH (%) = B × Nb/(α × Mp × h_tot_) × 100

where B is the consumption of the base (mL), Nb is the concentration of the base used (0.1 M), *α* is the average degree of the dissociation of the *α*-NH2 groups, Mp is the mass of SSP (g), and h_tot_ is the total number of peptide bonds in the protein substrate (6.088 mmol/g for SSP).

### 2.5. Inhibitory Activities Against Hyperglycemic Enzymes

The method of Sadeghi et al. [19] was referred to and modified slightly for *α*-glucosidase inhibition assay. *α*-Glucosidase and its substrate *p*-NPG was separately dissolved with PBS buffer (0.2 M, pH 6.8) to a concentration of 0.2 U/mL and 10 mM, respectively. A mixture of SSPH (100 μL) and *α*-glucosidase solution (50 μL) was added into 96-well plates and incubated at 37 °C for 15 min. Then, *p*-NPG solution (50 μL) was added, and the mixture was incubated at 37 °C for an additional 30 min. Finally, the absorbance was recorded at 405 nm.

The method of Wang et al. [20] was referred to and modified slightly for the DPP-IV inhibition assay. DPP-IV and its substrate Gly-Pro-*p*-nitroanilide were separately dissolved with Tris-HCl buffer (0.1 M, pH 8.0) to concentrations of 10 U/L and 1.6 mM, respectively. A mixture of SSPH (50 μL) and Gly-Pro-*p*-nitroanilide solution (50 μL) was added into 96-well plates and incubated at 37 °C for 10 min. Then, DPP-IV solution (100 μL) was added, and the mixture was incubated at 37 °C for 60 min. Finally, the absorbance was recorded at 405 nm.

The method of Huang et al. [21] was referred to and modified slightly for the *α*-amylase inhibition assay. *α*-amylase was dissolved with PBS buffer (0.1 M, pH 7.0) to a concentration of 1 U/mL. A mixture of SSPH (100 μL) and *α*-amylase solution (100 μL) was incubated at 37 °C for 10 min. Then, 200 μL soluble starch solution (1% *w*/*v*) was added, and the mixture was incubated at 37 °C for 10 min. Finally, 500 μL DNS reagent was introduced, and the reaction was conducted in a boiling water bath for 5 min, followed by rapid cooling for 5 min. The absorbance was recorded at 540 nm.

The inhibition was calculated by following formula:

$$enzyme\;inhibition\;(\%)=(1-\frac{A_{1}-A_{2}}{A_{3}-A_{4}})\times 100$$

where A_1_ is the absorbance of the sample group, A_2_ is that of the background group (without enzyme), A_3_ is that of the control group (without sample), and A_4_ is that of the control background group (without enzyme and sample). Corresponding buffer was added to ensure equal volumes across groups.

### 2.6. Process Optimization

The optimal enzyme was selected in foregoing screening experiment, which produced SSPH demonstrating the greatest potential with multiple inhibitory activities against hyperglycemic enzymes. A process based on the optimal enzyme was optimized using dual indices, i.e., *α*-glucosidase inhibition and DPP-IV inhibition. They represent two distinct mechanisms of blood glucose control through glycogen metabolism and hormone regulation, respectively [11,12].

Single-factor experiments were conducted to evaluate the impact of each factor on *α*-glucosidase inhibition and DPP-IV inhibition of the resultant SSPHs. The factors investigated included the concentration of SSP (ranging from 1% to 5%), enzyme dosage (ranging from 6000 to 14,000 U), and hydrolysis time (ranging from 60 to 180 min). The pH and temperature for hydrolysis were maintained as required by the optimal enzyme.

A Box–Behnken-Design Response Surface Methodology (BBD-RSM) experiment was performed to optimize the preparation conditions for achieving the best inhibition of *α*-glucosidase and DPP-IV by SSPH. Meanwhile, the interactions among different factors were also investigated [22]. According to the results for individual factors, a three-factor, three-level BBD-RSM design (shown in Table 1) was developed, comprising 17 tests, including five central point replicates (detailed in Table 2 and Table 3).

Following modeling and data analysis, the optimal preparation conditions were identified. Validation tests were conducted three times under the specified optimal conditions, and the results were compared to the predicted values derived from the corresponding model.

### 2.7. Peptide Identification

The peptide sequences in the optimized SSPH were identified at Shanghai Omicsolution Co., Ltd. (Shanghai, China). The SSPH was desalted by a Pierce C18 Spin Tip, and then dissolved in 0.1% formic acid solution. The solution was analyzed by Orbitrap Fusion Lumos coupled to an EASY-nanoLC 1200 system (Thermo Fisher Scientific, Waltham, MA, USA). A 5 μL peptide sample was loaded onto a 25 cm analytical column (75 μm inner diameter, 1.9 μm resin (Dr Maisch)) and separated with 60 min gradient starting at 4% buffer B (80% ACN with 0.1% FA) followed by a stepwise increase to 50% in 53.6 min, 95% in 40 s, and held for 5.6 min. The column flow rate was maintained at 300 nL/min with a column temperature of 40 °C. The electrospray voltage was set to 2 kV.

The mass spectrometer was run under data-dependent acquisition (DDA) mode, and automatically switched between MS and MS/MS mode. The survey of full-scan MS spectra (*m*/*z*: 100–1500) was acquired in Orbitrap with a 120,000 resolution, normalized automatic gain control (AGC) target of 200%, and maximum injection time of 50 ms. Then, the precursor ions were selected into collision cell for fragmentation by higher-energy collision dissociation (HCD), the normalized collection energy was 25%, 30%, and 35%; the MS/MS resolution was set at 50,000; the normalized automatic gain control (AGC) target was 200%; the maximum injection time was 25 ms; and the dynamic exclusion was 30 s.

Tandem mass spectra were processed by PEAKS Studio version 10.6 (Bioinformatics Solutions Inc., Waterloo, ON, Canada). The database was *Hippophae rhamnoides* (version 2024, 470 entries), which was downloaded from UniProt. None was set as the digestion enzyme. PEAKS DB were searched with a fragment ion mass tolerance of 0.02 Da and a parent ion tolerance of 10ppm. Oxidation on methionine and deamidation on asparagine and glutamine were specified as the variable modifications. The peptides with- 10lgP ≥ 20 and the proteins containing at least 1 unique peptide were filtered. The FDR (False Discovery Rate) filter threshold was 1%.

### 2.8. Bioinformatics Analysis

The peptide sequences were screened by several databases. Previously reported bioactivities were identified by BIOPEP. The bioactivity of sequences was predicted by PeptideRanker, with higher scores indicating greater potential. The protein sources of the peptides were identified by UniProt, while toxicity and allergenicity were predicted by ToxinPred and AllerTOP, respectively. The databases used are listed in Table 4. The visit dates were all before 30 June 2024.

### 2.9. Molecular Docking

The three-dimensional structures of *α*-glucosidase (PDB ID: 3WY1) and DPP-IV (PDB ID: 5J3J) were sourced from the PDB database on 28 May 2024 (website shown in Table 4). The three-dimensional structures of the peptides were generated by Chem Draw 22. Prior to docking, the peptides were designated as ligands, while *α*-glucosidase and DPP-IV were designated as receptors in AutoDockTools-1.5.7. For ligand preparation, hydrogen atoms were added, and the number of charges and the atomic rigidity properties were determined. Receptor preparation involved the removal of water molecules and the addition of hydrogen atoms. Docking simulations were performed to estimate the binding energy; lower binding energy values indicate more stable ligand–receptor complexes. The docking results were visualized by PyMOL 2.5.7, allowing for the assessment of binding interactions and the identification of potential binding sites within the enzymatic structures.

### 2.10. Solid-Phase Synthesis of Peptides

The peptides (purity ≥ 95%) were synthesized using the Fmoc solid-phase synthesis method (Sangon Biotech Co., Ltd., Shanghai, China).

### 2.11. Statistical Analysis

All experiments were conducted in triplicate, and the results were expressed as mean ± standard deviation. The BBD-RSM experiment was designed and analyzed by Design Expert 13. IC_50_ values were calculated with GraphPad Prism 8. Significance analyses were performed by IBM SPSS Statistics 26, with a *p*-value < 0.05 considered statistically significant.

## 3. Results and Discussion

### 3.1. Screening of Proteases

**Figure 1:**
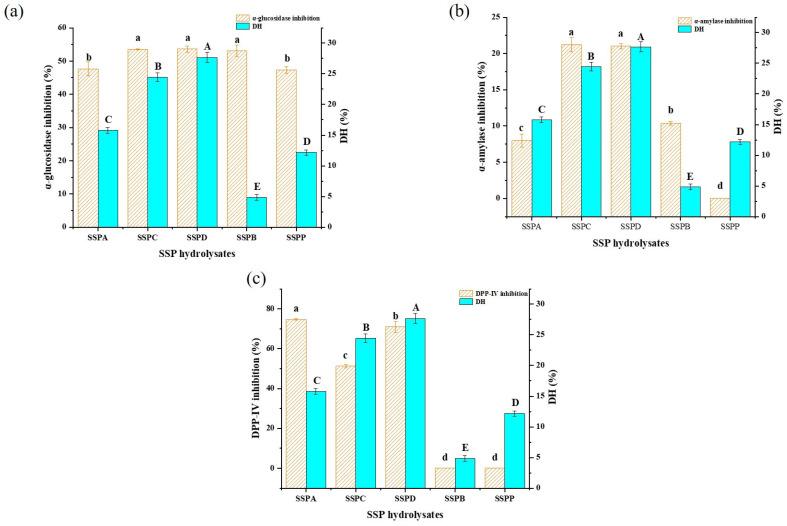
The DH and *α*-glucosidase inhibitions of SSPHs (**a**); the DH and *α*-amylase inhibitions of SSPHs (**b**); the DH and DPP-IV inhibitions of SSPHs (**c**). SSPA, SSPC, SSPD, SSPB, and SSPP stand for SSP hydrolyzed by alkaline proteinase, compound proteinase, dispase, bromelain, and papain. Uppercase and lowercase letters are the significance analysis result of DH and inhibitions by IBM SPSS Statistics, respectively.

The DH of SSPHs and the inhibitions of SSPHs against α-glucosidase, α-amylase, and DPP-IV are shown in Figure 1a–c, respectively. The DH results from high to low were SSPHD (27.69 ± 0.79%), SSPHC (24.45 ± 0.70%), SSPHA (15.78 ± 0.47%), SSPHP (12.18 ± 0.43%), and SSPHB (4.85 ± 0.46%). Among them, dispase hydrolyzed SSP most effectively, which might be related to its specific action on aliphatic amino acids and aromatic amino acids. The content of related amino acids in SSP was 18.33 g/g, accounting for 22.67% of the amino acid composition. Similarly, the effects of other proteases were not as good as that of dispase. This is mainly because the action sites of other proteases are fewer in the SSP or they cannot bind to it as easily. The inhibition results indicate that the SSPHs pertaining to different proteases exhibited significant differences in inhibitions. Specifically, SSP hydrolyzed by dispase (SSPD) demonstrated the strongest inhibitory effect on α-glucosidase (53.60 ± 0.98%). For α-amylase, SSP hydrolyzed by compound proteinase exhibited the highest inhibitory effect (21.22 ± 0.97%), closely followed by dispase (21.01 ± 0.38%). Statistical analysis revealed no significant difference between the two values (*p* > 0.05). For DPP-IV, SSP hydrolyzed by alkaline proteinase exhibited the strongest inhibitory effect (74.44 ± 0.44%), followed by dispase (70.98 ± 2.74%). SSPD demonstrated overall inhibitory effects across all three enzymes, making it a promising candidate for hypoglycemic activity. Moreover, the DH of SSPD was the highest among the SSPHs prepared by means of the proteases tested. This may explain why SSPD has a potential impact on hypoglycemic activity, as it may release a greater variety and quantity of peptides that possess one or more inhibitions [23].

### 3.2. Single-Factor Experiments

Both *α*-glucosidase and *α*-amylase are crucial enzymes that regulate the rate at which complex carbohydrates are broken down into absorbable monosaccharides. In single-factor experiments, *α*-glucosidase was chosen as the representative enzyme for investigating blood glucose control through glycogen metabolism, while DPP–IV was selected to represent blood glucose regulation via hormonal mechanisms. The objective was to optimize the preparation of SSPD to maximize its impact on these distinct regulatory pathways. Consequently, during the process optimization and subsequent experiments, our primary focus was on evaluating the inhibitory effects on *α*-glucosidase and DPP-IV activities.

**Figure 2:**
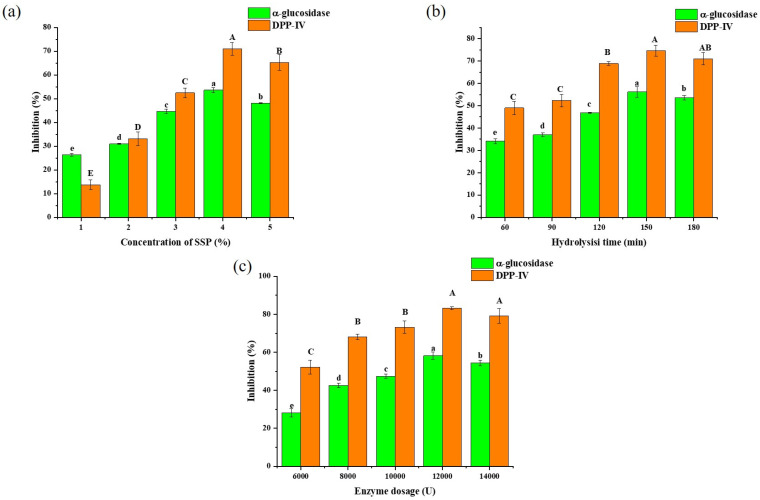
Effect of concentration of SSP on the *α*-glucosidase and DPP-IV inhibitory activity of SSPD (**a**). Effect of hydrolysis time on the *α*-glucosidase and DPP-IV inhibitory activity of SSPD (**b**). Effect of enzyme dosage on the *α*-glucosidase and DPP-IV inhibitory activity of SSPD (**c**). Uppercase and lowercase letters are the significance analysis result of *α*-glucosidase and DPP-IV inhibitory activity of SSPD by IBM SPSS Statistics, respectively.

To assess the impact of SSP concentration (Figure 2a), concentrations of 1%~5% were tested, while keeping the enzyme dosage (8400 U) and hydrolysis time (180 min) constant. As the concentration increased from 1% to 4%, the inhibition of *α*-glucosidase and DPP-IV significantly increased, reaching 53.60 ± 0.98% and 70.98 ± 2.74%, respectively. However, when the concentration was further elevated to 5%, a decreasing trend in inhibition was observed. Consequently, 4% was identified as the optimal concentration of SSP.

To evaluate the impact of hydrolysis time (Figure 2b), inhibitions were measured at 30 min intervals from 60 to 180 min. As the hydrolysis time increased from 60 to 150 min, the inhibitions increased significantly, reaching 56.13 ± 2.32% and 74.67 ± 2.40%, respectively.

However, when the hydrolysis time exceeded 150 min, the inhibitions declined slightly, suggesting that active peptides were further degraded [24]. Consequently, 150 min was identified as the optimal hydrolysis time.

To assess the influence of enzyme dosage (Figure 2c), experiments were conducted from 6000 to 14,000 U. When the enzyme dosage was increased from 6000 to 12,000 U, both inhibitions increased significantly, reaching 58.17 ± 1.93% and 83.15 ± 0.70%, respectively. This was due to the addition of more enzymes, which accelerated the enzymatic hydrolysis process, resulting in a more complete and rapid hydrolysis [25]. However, when the enzyme dosage exceeded 12,000 U, the inhibitions gradually reached a maximum, followed by a slight decrease. This phenomenon was likely due to the excessive addition of enzymes, leading to a greater hydrolysis of SSP into free amino acids [26]. Consequently, 12,000 U was identified as the optimal enzyme dosage.

### 3.3. BBD-RSM Experiments

**Figure 3:**
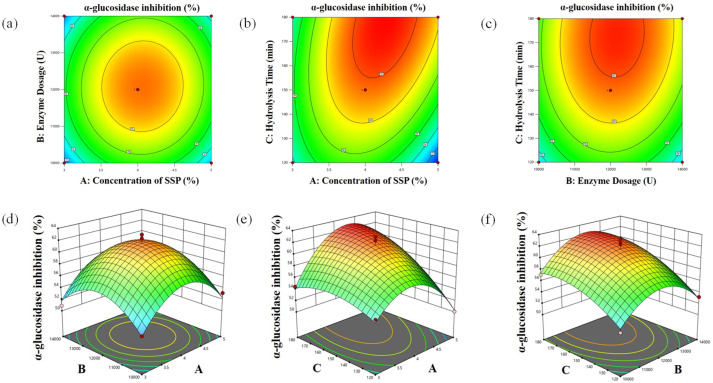
The contour plots (**a**–**c**) and 3D response surface plots (**d**–**f**) of *α*-glucosidase inhibition.

**Figure 4:**
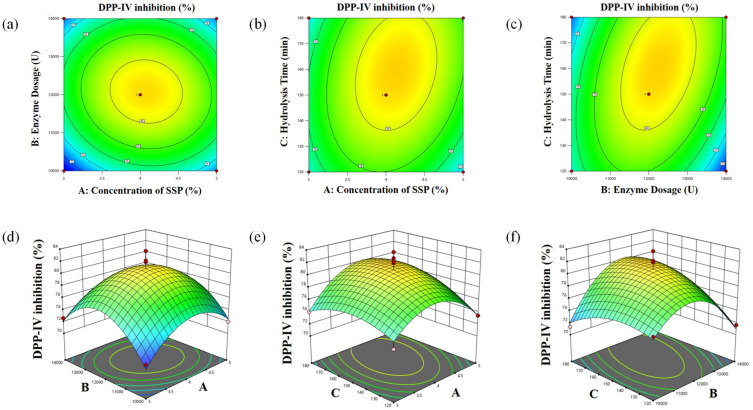
The contour plots (**a**–**c**) and 3D response surface plots (**d**–**f**) of DPP-IV inhibition.

The findings from the BBD-RSM regarding *α*-glucosidase and DPP-IV inhibition are presented in Table 2 and Table 3, respectively. The contour plots and three-dimensional response surfaces depicted in Figure 3 and Figure 4 were utilized to demonstrate how interactions among the independent variables influence the response values [27]. The quadratic polynomial equation derived from the linear regression analysis, with *α*-glucosidase inhibition designated as the experimental response, can be expressed as follows:

Y = 61.56 + 0.66X_1_ + 0.37X_2_ + 2.64X_3_ + 0.22X_1_X_2_ + 2.38X_1_X_3_ + 0.50X_2_X_3_ − 5.17X_1_^2^ − 4.12X_2_^2^ − 1.74X_3_^2^

The quadratic polynomial regression model for *α*-glucosidase inhibition (as shown in Table 5) revealed that the linear coefficient C, alongside the quadratic and cross coefficients AC, are statistically significant (*p* < 0.05), while the other coefficients (A, B, AB, BC) do not hold significance. The *p*-value for the “Model” term is below 0.0001, indicating that the model is significant and demonstrates good regression; conversely, the “Lack of Fit” term with *p* = 0.5951 (>0.05) is not significant, suggesting the model fits well. The R^2^ = 0.9726 indicates a robust correlation between the variables, which facilitates precise predictions of the values of the independent variable within the parameters set during the experiments. The adjusted R^2^ = 0.9374 and predicted R^2^ = 0.8201 reveal a minimal difference of less than 0.2. This small discrepancy reinforces the adequacy of the quadratic model in fitting the data well and highlights its effectiveness in representing the actual processes observed in the study. The coefficient of variation (C.V.) is 1.95, which serves as an affirmation of the model’s precision and trustworthiness. A low C.V. indicates a reliable model with minimal relative variability, enhancing confidence in the predictions made. Additionally, the adequate precision value is 13.3485 (>4), which is also deemed acceptable.

The quadratic polynomial equation derived from the linear regression analysis, with DPP-IV inhibition designated as the experimental response, can be expressed as follows:

Y = 80.86 + 0.65X_1_ + 0.48X_2_ + 1.00X_3_ − 0.58X_1_X_2_ + 1.25X_1_X_3_ + 2.35X_2_X_3_ − 3.97X_1_^2^ − 5.16X_2_^2^ − 1.88X_3_^2^

In the regression model examining DPP-IV inhibition (refer to Table 6), both quadratic coefficients A^2^ and B^2^ demonstrate significance (*p* < 0.05), while the other coefficients are not. The *p*-value is lower than 0.0001, indicating strong significance, and the “Lack of Fit” term shows no significant influence. The model exhibits an R^2^ value of 0.8953, indicating a strong correlation. The adjusted R^2^ and predicted R^2^ for the model are 0.7607 and 0.5656, respectively, revealing a variation of 0.2. Furthermore, the C.V. value and adequate precision are 2.73 and 6.4876, respectively, both falling within acceptable limits.

In conclusion, the analysis confirms that the constructed quadratic model is statistically valid and dependable, suitable for optimizing SSPD preparation. From the regression outcomes of the BBD-RSM experiments, the projected optimal parameters identified are as follows: SSP concentration 4.14%, enzyme dosage 12,279.10 U, and hydrolysis time 138.51 min. For practical application, the optimal parameters are rounded as follows: SSP concentration 4%, enzyme dosage 12,300 U, and hydrolysis time 140 min, under which the experimental measurements of *α*-glucosidase inhibition and DPP-IV inhibition were found to be 58.73 ± 2.25% and 76.53 ± 2.74%, respectively. The actual outcomes closely align with the predictions from the theoretical quadratic model, demonstrating relative error rates of 1.37% and 3.44%, respectively, thereby reaffirming the model’s high precision and efficacy in accurately reflecting the actual preparation conditions.

### 3.4. Identification by Peptidomics and Bioinformatics Analysis

A sum of 1093 peptides were identified through a peptidomic analysis of SSPD, including IGL and APG, which have previously been reported as DPP-IV inhibitors. Among these 1093 peptides, 76 potential bioactive sequences were selected for *α*-glucosidase inhibition based on the following rules [28]: 1. Sequences were unmodified by functional groups; 2. Sequences had a bioactivity score higher than 0.8; 3. Sequences were non-toxic and non-allergenic; 4. Sequences were derived from proteins rather than other enzyme sources; 5. Sequences had not been previously reported; 6. Sequences had a peptide chain length less than 15 and a peak area over 1 × 10^5^.

For DPP-IV inhibition, the 76 peptides were further screened for the presence of Ala or Pro at the second position of the −NH_2_ terminus and Leu or Ile at the −NH_2_ terminus [29,30]. Under these conditions, 30 potential bioactive sequences were selected. Detailed information regarding the peptides is provided in the Supplementary Materials.

### 3.5. Molecular Docking Analysis

To further evaluate the aforementioned peptides, molecular docking was employed to investigate the autonomous binding capabilities of 76 screened peptides to *α*-glucosidase and 30 peptides to DPP-IV. The binding energies for the majority of the peptides were negative, including 71 of 76 peptides and all 30 peptides (as detailed in the Supplementary Materials). These findings suggest that the binding processes of these peptides were autonomous, indicating their potential as inhibitors of *α*-glucosidase and DPP-IV, respectively. Notably, the reference DPP-IV inhibitors IGL and APG exhibited binding energies of −3.75 kcal/mol and −3.78 kcal/mol, respectively. Several screened peptides demonstrated even lower binding energies. Specifically, FFI, FGI, and FHF exhibited α-glucosidase binding energies below −4.40 kcal/mol, suggesting more stable complexes and potentially stronger inhibitory effects. Similarly, LFF, IGF, and IYF showed DPP-IV binding energies below −4.15 kcal/mol, indicating high binding affinity.

**Figure 5:**
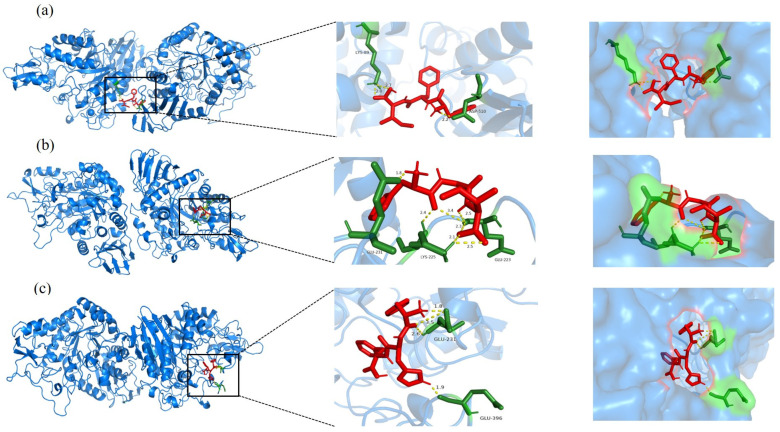
Molecular docking diagram of screened peptides (**a**) FFI, (**b**) FGI, and (**c**) FHF with *α*-glucosidase. (The 3D red stick models are peptides. The green stick models are *α*-glucosidase residues. Hydrogen bonds are shown with a yellow dotted line).

The docking interactions between *α*-glucosidase and the peptides showed that FFI, FGI, FHF, and FFI formed two hydrogen bonds with the *α*-glucosidase residues LYS89 and ASP510; similarly, FHF formed two hydrogen bonds with the *α*-glucosidase residues GLU231 and GLU396; and FGI formed three hydrogen bonds with the *α*-glucosidase residues GLU223, LYS225, and GLU231 (Figure 5).

**Figure 6:**
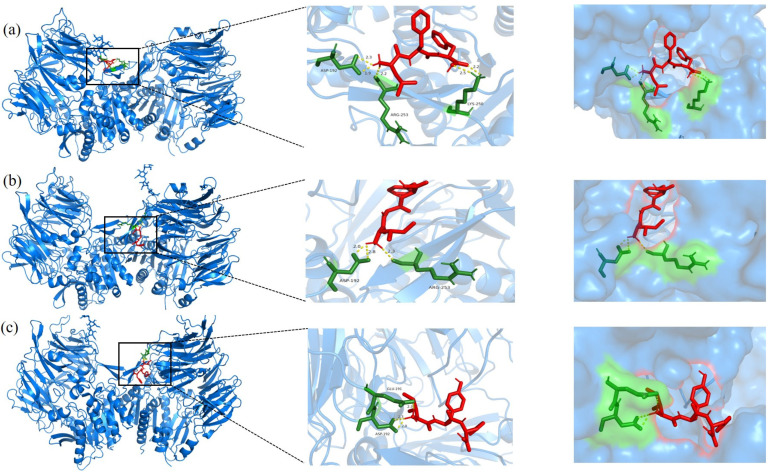
Molecular docking diagram of screened peptides (**a**) LFF, (**b**) IGF, and (**c**) IYF with DPP-IV. (The 3D red stick models are peptides. The green stick models are DPP-IV residues. Hydrogen bonds are shown with a yellow dotted line).

The docking interactions between DPP-IV and the peptides demonstrated that LFF, IGF, IYF, and LFF formed three hydrogen bonds with the DPP-IV residues ASP192, LYS250, and ARG253; IGF formed two hydrogen bonds with the DPP-IV residues ASP192 and ARG253; and, similarly, IYF formed two hydrogen bonds with DPP-IV residues GLU191 and ASP192 (Figure 6).

The aforementioned molecular docking findings suggest that the six SSMP-derived antihyperglycemic peptides could potentially obstruct the function of *α*-glucosidase or DPP-IV by attaching to one or several domains or active sites.

### 3.6. In Vitro Activity Verification of Peptides

The three potential α-glucosidase inhibitors and three potential DPP-IV inhibitors, which were identified in Section 3.5, were synthesized and assessed in vitro for their inhibitory capacity against α-glucosidase and DPP-IV, with the results presented in Table 7 and Table 8, respectively. Meanwhile, SSPD purification, which was prepared by hydrolysis under optimal conditions, dialysis (200 Da), ultrafiltration (3000 Da), and freeze-drying, was also evaluated in vitro for its inhibitory capacity. Acarbose and Diprotin A (IPI) served as positive controls for *α*-glucosidase and DPP-IV inhibition in vitro, respectively. The IC_50_ values for Acarbose and IPI were 0.80 ± 0.04 mM and 8.57 ± 0.40 μM, which are consistent with the values reported by Kawakami et al. [31] and Nongoniermail et al. [29]. For *α*-glucosidase, FHF exhibited the lowest IC_50_ value (3.98 ± 0.16 mM), followed by FFI (IC_50_: 8.21 ± 0.21 mM) and FGI (IC_50_: 11.57 ± 0.20 mM). For DPP-IV, IYF exhibited the lowest IC_50_ value (5.32 ± 0.15 mM), followed by IGF (IC_50_: 7.17 ± 0.14 mM) and LFF (IC_50_: 7.62 ± 0.19 mM). These IC_50_ results confirm that the screened peptides possess specific inhibitory activities against *α*-glucosidase and DPP-IV. The IC_50_ values of SSPD against *α*-glucosidase and DPP-IV were 3.45 ± 0.18 mg/mL and 5.01 ± 0.21 mg/mL, respectively, similar to those of highly purified inhibitory peptides [32,33].

## 4. Conclusions

The study found that SSPD exhibited simultaneous inhibitory abilities against *α*-glucosidase, *α*-amylase, and DPP-IV. Under optimal conditions (SSP concentration: 4%, enzyme dosage: 12,300 U, hydrolysis time: 140 min), the IC_50_ values of SSPD purification for *α*-glucosidase and DPP-IV were 3.45 ± 0.18 mg/mL and 5.01 ± 0.21 mg/mL, respectively. Through peptidomic analysis, peptide sequence screening, and molecular docking validation, three potential *α*-glucosidase inhibitors (FHF, FFI, FGI) and three potential DPP-IV inhibitors (IYF, IGF, LFF) were identified. All six peptides demonstrated varying degrees of inhibition against *α*-glucosidase or DPP-IV, confirmed by in vitro activity assays. Ranking by activity, the *α*-glucosidase inhibitory peptides was in the order FHF (IC_50_: 3.98 ± 0.16 mM) > FFI (IC_50_: 8.21 ± 0.21 mM) > FGI (IC_50_: 11.57 ± 0.20 mM), while the DPP-IV inhibitory peptides was in the order IYF (IC_50_: 5.32 ± 0.15 mM) > IGF (IC_50_: 7.17 ± 0.14 mM) > LFF (IC_50_: 7.62 ± 0.19 mM). Therefore, SSPD holds significant promise for the development and application of natural hypoglycemic peptide, contributing to the reduction in waste from SSP resources and enhancing the added value of seabuckthorn seed meal. In the future, in vitro digestion experiments and animal experiments will be conducted to further evaluate the hypoglycemic effect of SSPD.

## Figures and Tables

**Table 1:** Factors and levels of Box–Behnken experimental design for α-glucosidase inhibition and DPP-IV inhibition.

Factor	Levels		
	−1	0	1
A: Concentration of SSP (%)	3	4	5
B: Enzyme dosage (U)	10,000	12,000	14,000
C: Hydrolysis time (min)	120	150	180

**Table 2:** Results of Box–Behnken experiments for α-glucosidase inhibition.

Std	Run	A: Concentration of SSP (%)	B: Enzyme Dosage (U)	C: Hydrolysis Time (min)	α-Glucosidase Inhibition (%)
15	1	0	0	0	62.98 ± 0.96
9	2	0	−1	−1	52.54 ± 0.97
8	3	1	0	1	59.80 ± 1.66
2	4	1	−1	0	53.12 ± 1.12
6	5	1	0	−1	50.07 ± 0.85
14	6	0	0	0	62.35 ± 1.11
3	7	−1	1	0	50.89 ± 0.82
4	8	1	1	0	53.37 ± 1.41
11	9	0	−1	1	57.12 ± 1.73
16	10	0	0	0	61.46 ± 2.44
1	11	−1	−1	0	51.50 ± 0.74
7	12	−1	0	1	54.48 ± 1.37
5	13	−1	0	−1	54.25 ± 1.57
12	14	0	1	1	59.77 ± 1.48
10	15	0	1	−1	53.18 ± 0.35
13	16	0	0	0	59.94 ± 1.05
17	17	0	0	0	61.05 ± 1.37

Data are presented as mean ± SD, *n* = 3.

**Table 3:** Results of Box–Behnken experiments for DPP-IV inhibition.

Std	Run	A: Concentration of SSP (%)	B: Enzyme Dosage (U)	C: Hydrolysis Time (min)	DPP-IV Inhibition (%)
15	1	0	0	0	78.25 ± 1.62
9	2	0	−1	−1	75.13 ± 1.01
8	3	1	0	1	79.05 ± 2.33
2	4	1	−1	0	72.01 ± 1.44
6	5	1	0	−1	73.44 ± 1.26
14	6	0	0	0	81.92 ± 1.73
3	7	−1	1	0	72.62 ± 0.64
4	8	1	1	0	71.58 ± 2.02
11	9	0	−1	1	71.31 ± 2.26
16	10	0	0	0	83.69 ± 2.76
1	11	−1	−1	0	70.72 ± 0.96
7	12	−1	0	1	74.06 ± 2.13
5	13	−1	0	−1	73.47 ± 2.21
12	14	0	1	1	77.20 ± 1.80
10	15	0	1	−1	71.62 ± 1.12
13	16	0	0	0	78.25 ± 2.46
17	17	0	0	0	82.17 ± 1.71

Data are presented as mean ± SD, *n* = 3.

**Table 4:** Database information used in bioinformatics analysis and molecular docking.

Database	Web Link
BIOPEP	https://biochemia.uwm.edu.pl/ (accessed on 17 May 2024)
PeptideRanker	http://distilldeep.ucd.ie/PeptideRanker/ (accessed on 18 May 2024).
UniProt	https://www.uniprot.org/ (accessed on 10 May 2024).
ToxinPred	https://webs.iiitd.edu.in/raghava/toxinpred/index.html (accessed on 19 May 2024).
AllerTOP	https://www.ddg-pharmfac.net/allertop_test (accessed on 19 May 2024).
PDB	https://www.rcsb.org (accessed on 3 June 2024).

**Table 5:** Analysis of variance for Box–Behnken experiments for α-glucosidase inhibition.

Source	Sum of Squares	df	Mean Square	F-Value	*p*-Value	
Model	301.32	9	33.48	27.64	0.0001	significant
A: Concentration of SSP	3.43	1	3.43	2.83	0.1362	
B: Enzyme dosage	1.07	1	1.07	0.8860	0.3779	
C: Hydrolysis time	55.81	1	55.81	46.80	0.0003	
AB	0.1849	1	0.1849	0.1527	0.7076	
AC	22.56	1	22.56	18.63	0.0035	
BC	1.01	1	1.01	0.8339	0.3915	
A^2^	112.51	1	112.51	92.89	< 0.0001	
B^2^	73.10	1	73.10	60.35	0.0001	
C^2^	12.70	1	12.70	10.49	0.0143	
Residual	8.48	7	1.21			
Lack of Fit	2.94	3	0.9812	0.7091	0.5951	not significant
Pure Error	5.53	4	1.38			
Cor Total	309.80	16				
R^2^	0.9726					
Adjusted R^2^	0.9374					
Predicted R^2^	0.8201					
Adeq Precision	13.3485					
Std. Dev.	1.10					
Mean	56.35					
C.V. %	1.95					

**Table 6:** Analysis of variance for Box–Behnken experiments design for DPP-IV inhibition.

Source	Sum of Squares	df	Mean Square	F-Value	*p*-Value	
Model	254.93	9	28.33	6.65	0.0103	significant
A: Concentration of SSP	3.39	1	3.39	0.7967	0.4017	
B: Enzyme dosage	1.85	1	1.85	0.4350	0.5306	
C: Hydrolysis time	7.92	1	7.92	1.86	0.2149	
AB	1.36	1	1.36	0.3187	0.5900	
AC	6.30	1	6.30	1.48	0.2633	
BC	22.09	1	22.09	5.19	0.0569	
A^2^	66.25	1	66.25	15.56	0.0056	
B^2^	111.97	1	111.97	26.29	0.0014	
C^2^	14.95	1	14.95	3.51	0.1031	
Residual	29.81	7	4.26			
Lack of Fit	5.34	3	1.78	0.2910	0.8308	not significant
Pure Error	24.47	4	6.12			
Cor Total	284.75	16				
R^2^	0.8953					
Adjusted R^2^	0.7607					
Predicted R^2^	0.5656					
Adeq Precision	6.8476					
Std. Dev.	2.06					
Mean	75.68					
C.V. %	2.73					

**Table 7:** In vitro IC_50_ of α-glucosidase inhibitory peptides. The use of ^a–d^ are the significance analysis result of *α*-glucosidase inhibition IC_50_ between *α*-glucosidase inhibitory peptides by IBM SPSS Statistics.

Sequence/Name	α-Glucosidase Inhibition IC_50_^a^ (mg/mL)	α-Glucosidase Inhibition IC_50_^a^ (mM)
FHF	1.79 ± 0.07 ^c^	3.98 ± 0.16 ^c^
FFI	3.50 ± 0.09 ^b^	8.21 ± 0.21 ^b^
FGI	3.88 ± 0.07 ^a^	11.57 ± 0.20 ^a^
SSPD-purification	3.45 ± 0.18 ^b^	-
Acarbose	0.52 ± 0.03 ^d^	0.80 ± 0.04 ^d^

**Table 8:** In vitro IC_50_ of DPP-IV inhibitory peptides. The use of ^a–d^ are the significance analysis result of DPP-IV inhibition IC_50_ between DPP-IV inhibitory peptides by IBM SPSS Statistics.

Sequence/Name	DPP-IV Inhibition IC_50_^a^ (mg/mL)	DPP-IV Inhibition IC_50_^a^ (mM)
IYF	2.35 ± 0.07 ^c^	5.32 ± 0.15 ^c^
IGF	2.40 ± 0.05 ^c^	7.17 ± 0.14 ^b^
LFF	3.24 ± 0.08 ^b^	7.62 ± 0.19 ^a^
SSPD-purification	5.01 ± 0.21 ^a^	-
IPI	3.91 × 10^−3^ ± 0.18 ^d^	8.57 × 10^−3^ ± 0.40 ^d^

## Data Availability

The original contributions presented in this study are included in the article/Supplementary Materials. Further inquiries can be directed to the corresponding author.

## Supplementary Materials

The following supporting information can be downloaded at: https://www.mdpi.com/article/10.3390/foods14111876/s1.

## References

[1] Ma Y., Yao J., Zhou L., Zhao M., Wang W., Liu J., Marchioni E. (2024). Comprehensive untargeted lipidomic analysis of sea buckthorn using UHPLC-HR-AM/MS/MS combined with principal component analysis. Food Chem..

[2] Dong K., Binosha Fernando W.M.A.D., Durham R., Stockmann R., Jayasena V. (2021). Nutritional Value, Health-promoting Benefits and Food Application of SeaBuckthorn. Food Rev. Int..

[3] Tkacz K., Turkiewicz I.P., Nowicka P., Wojdyło A. (2024). Microspheres as carriers of seabuckthorn carotenoids and tocols with antidiabetic potential: Effect of biopolymers, cross-linking and storage. Food Biosci..

[4] Chen A., Feng X., Dorjsuren B., Chimedtseren C., Damda T.-A., Zhang C. (2023). Traditional food, modern food and nutritional value of Seabuckthorn (*Hippophae rhamnoides* L.): A review. J. Future Foods.

[5] Lin J., Xiang H., Sun-Waterhouse D., Cui C., Wang W. (2022). Deep eutectic solvents and alkaline extraction of protein from seabuckthorn seed meal: A comparison study. Food Sci. Hum. Wellness.

[6] Prandi B., Faccini A., Lambertini F., Bencivenni M., Jorba M., Van Droogenbroek B., Bruggeman G., Schober J., Petrusan J., Elst K. (2019). Food wastes from agrifood industry as possible sources of proteins: A detailed molecular view on the composition of the nitrogen fraction, amino acid profile and racemisation degree of 39 food waste streams. Food Chem..

[7] Qin X., Zhang T., Cao Y., Deng B., Zhang J., Zhao J. (2020). Effects of dietary seabuckthorn pomace supplementation on skeletal muscle mass and meat quality in lambs. Meat Sci..

[8] Vaiserman A., Lushchak O. (2019). Developmental origins of type 2 diabetes: Focus on epigenetics. Ageing Res. Rev..

[9] Roden M., Shulman G.I. (2019). The integrative biology of type 2 diabetes. Nature.

[10] Yu Z., Yin Y., Zhao W., Yu Y., Liu B., Liu J., Chen F. (2011). Novel peptides derived from egg white protein inhibiting alpha-glucosidase. Food Chem..

[11] Patil P., Mandal S., Tomar S.K., Anand S. (2015). Food protein-derived bioactive peptides in management of type 2 diabetes. Eur. J. Nutr..

[12] Lacroix I.M.E., Li-Chan E.C.Y. (2016). Food-derived dipeptidyl-peptidase IV inhibitors as a potential approach for glycemic regulation-Current knowledge and future research considerations. Trends Food Sci. Technol..

[13] Mojica L., de Mejia E.G. (2016). Optimization of enzymatic production of anti-diabetic peptides from black bean (*Phaseolus vulgaris* L.) proteins, their characterization and biological potential. Food Funct..

[14] Wang F., Yu G., Zhang Y., Zhang B., Fan J. (2015). Dipeptidyl Peptidase IV Inhibitory Peptides Derived from Oat (*Avena sativa* L.), Buckwheat (*Fagopyrum esculentum*), and Highland Barley (*Hordeum vulgare trifurcatum* (L.) Trofim) Proteins. J. Agric. Food Chem..

[15] Xiang H., Waterhouse D.-S., Liu P., Waterhouse G.I.N., Li J., Cui C. (2020). Pancreatic lipase-inhibiting protein hydrolysate and peptides from seabuckthorn seed meal: Preparation optimization and inhibitory mechanism. LWT-Food Sci. Technol..

[16] Leeb E., Gotz A., Letzel T., Cheison S.C., Kulozik U. (2015). Influence of denaturation and aggregation of beta-lactoglobulin on its tryptic hydrolysis and the release of functional peptides. Food Chem..

[17] Zan R., Zhu L., Wu G., Zhang H. (2023). Identification of Novel Peptides with Alcohol Dehydrogenase (ADH) Activating Ability in Chickpea Protein Hydrolysates. Foods.

[18] Padial-Dominguez M., Espejo-Carpio F.J., Garcia-Moreno P.J., Jacobsen C., Guadix E.M. (2020). Protein derived emulsifiers with antioxidant activity for stabilization of omega-3 emulsions. Food Chem..

[19] Sadeghi M., Miroliaei M., Ghanadian M., Szumny A., Rahimmalek M. (2023). Exploring the inhibitory properties of biflavonoids on alpha-glucosidase; computational and experimental approaches. Int. J. Biol. Macromol..

[20] Wang L., Ding L., Du Z., Yu Z., Liu J. (2019). Hydrolysis and Transport of Egg White-Derived Peptides in Caco-2 Cell Monolayers and Everted Rat Sacs. J. Agric. Food Chem..

[21] Huang Y., Richardson S.J., Brennan C.S., Kasapis S. (2024). Mechanistic insights into α-amylase inhibition, binding affinity and structural changes upon interaction with gallic acid. Food Hydrocoll..

[22] Yolmeh M., Jafari S.M. (2017). Applications of Response Surface Methodology in the Food Industry Processes. Food Bioprocess Technol..

[23] Ashraf Z.U., Gani A., Shah A., Gani A. (2024). Identification of antidiabetic peptides from broad bean protein: Sequencing using LC-MS-QTOF and in-vitro confirmative studies. Food Biosci..

[24] Xu X., Liu W., Liu C., Luo L., Chen J., Luo S., McClements D.J., Wu L. (2016). Effect of limited enzymatic hydrolysis on structure and emulsifying properties of rice glutelin. Food Hydrocoll..

[25] Yuan G., Li W., Pan Y., Wang C., Chen H. (2018). Shrimp shell wastes: Optimization of peptide hydrolysis and peptide inhibition of α-amylase. Food Biosci..

[26] Zhang L., Wang Y., Ni Y., Zhang Y., Liu Y., Du B., Luo H., Lin D. (2024). Production of novel mung bean peptides-based zinc supplement: Process optimization, chelation mechanism, and stability assessment in vitro. Food Biosci..

[27] Fu X., Wang D., Belwal T., Xu Y., Li L., Luo Z. (2021). Sonication-synergistic natural deep eutectic solvent as a green and efficient approach for extraction of phenolic compounds from peels of *Carya cathayensis* Sarg. Food Chem..

[28] Zan R., Wu Q., Chen Y., Wu G., Zhang H., Zhu L. (2023). Identification of Novel Dipeptidyl Peptidase-IV Inhibitory Peptides in Chickpea Protein Hydrolysates. J. Agric. Food Chem..

[29] Nongonierma A.B., Cadamuro C., Le Gouic A., Mudgil P., Maqsood S., FitzGerald R.J. (2019). Dipeptidyl peptidase IV (DPP-IV) inhibitory properties of a camel whey protein enriched hydrolysate preparation. Food Chem..

[30] Nongonierma A.B., FitzGerald R.J. (2019). Features of dipeptidyl peptidase IV (DPP-IV) inhibitory peptides from dietary proteins. J. Food Biochem..

[31] Kawakami K., Li P., Uraji M., Hatanaka T., Ito H. (2014). Inhibitory effects of pomegranate extracts on recombinant human maltase-glucoamylase. J. Food Sci..

[32] Yu H., Xian M., Qu C., Peng P., Yongo E., Guo Z., Du Z., Xiao J. (2024). Novel Se-enriched α-glucosidase inhibitory peptide derived from tuna dark meat: Preparation, identification and effects on IR-HepG2 cells. Food Biosci..

[33] Ortizo R.G.G., Sharma V., Tsai M.-L., Nargotra P., Sun P.-P., Chen C.-W., Dong C.-D. (2024). A novel deep eutectic solvent-based green extraction and purification of DPP-IV inhibitory peptides from tilapia (*Oreochromis niloticus*) viscera hydrolysate. Food Biosci..

